# Torsional Resistance of Heat-Treated Nickel-Titanium Instruments under Different Temperature Conditions

**DOI:** 10.3390/ma14185295

**Published:** 2021-09-14

**Authors:** Hyo Jin Jo, Sang Won Kwak, Hyeon-Cheol Kim, Sung Kyo Kim, Jung-Hong Ha

**Affiliations:** 1 Department of Conservative Dentistry, School of Dentistry, Kyungpook National University, Daegu 41940, Korea; sallyes2004@naver.com (H.J.J.); skykim@knu.ac.kr (S.K.K.); 2 Department of Conservative Dentistry, School of Dentistry, Dental Research Institute, Pusan National University, Yangsan 50612, Korea; endokwak@pusan.ac.kr (S.W.K.); golddent@pusan.ac.kr (H.-C.K.)

**Keywords:** body temperature, C-wire, differential scanning calorimetry, electrical discharge machining, gold-wire, heat-treated nickel-titanium file, torsional resistance

## Abstract

This study compared the torsional resistance of heat-treated nickel-titanium (NiTi) instruments under different temperature conditions. Four thermomechanically treated single-use NiTi rotary instruments were selected for this study: OneShape (OS), OneCurve (OC), WaveOne Gold (WOG) and HyFlex EDM (HFE). Each instrument was further subdivided by temperature into 2 subgroups. Maximum torque and the distortion angle until fracture occurred were evaluated. Differential scanning calorimetry analysis was performed to measure the phase transformation temperature. Statistical analysis was performed using a two-way ANOVA and *t*-test (*p* < 0.05). Fractured fragments were observed using scanning electron microscopy (SEM). The two-way ANOVA showed no significant differences for different temperature conditions. At both room (RT) and body temperature (BT), OS was predominantly austenite while HFE was martensite. OC and WOG were predominantly martensite at RT and mixed phase at BT. At BT, more than half of WOG was martensite, while half of OC was austenite. SEM examination showed no topographical differences between instruments in different temperature groups. In relation to a limitation of this study, there was no difference in torsional resistance of NiTi rotary instruments between the BT and RT conditions. This implies that clinicians do not need to consider a decrease of torsional resistance of heat-treated NiTi instruments at BT.

## 1. Introduction

Over 30 years have passed since nickel-titanium (NiTi) rotary instruments were first introduced and became essential instruments in the endodontic field. Despite their attractive advantages, including superior flexibility, maintaining original curves of the root canal and subsequently minimizing iatrogenic complications, instrument separation is still considered a major concern. According to previous studies on NiTi rotary instrument separation, the main causes of fracture are cyclic fatigue fracture and torsional failure [1,2,3,4,5,6,7]. Cyclic fatigue fracture occurs when repeated tensile stress is concentrated on the NiTi instrument in the curved canal. However, torsional failure results from a locked instrument in a certain part of the root canal.

Many attempts have been made by engineers and clinicians to resolve these flaws. Various heat-treatment technologies, including M-wire, Blue-wire, Gold-wire, C-wire and the electrical discharge machining (EDM) process, have been introduced to enhance the metallurgical properties of NiTi alloys. M-wire, Blue-wire and Gold-wire were proven to improve fracture resistance and flexibility [8]. The manufacturer claims that C-wire technology could provide better flexibility and adaptivity for OneCurve [9]. Meanwhile, the EDM process is well-known as a noncontact machining procedure, using electrical discharge machining, that offers enhanced flexibility [8].

Heat treatment could change the phase transition temperature of the NiTi instrument, which changes its phase composition and the physical performance of the metal alloy under specific temperature conditions [8,9,10,11,12]. Although numerous studies reported the fracture resistance of heat-treated NiTi instruments, most of them were conducted under room temperature (RT) conditions. However, the environment that the instrument is used is at intraoral body temperature (BT), which has different temperature conditions compared with most in vitro test conditions; this difference might affect its mechanical performance due to the altered composition of phase.

Previous studies of cyclic fatigue tests were conducted under different temperature conditions and showed different cyclic fatigue resistance depending on temperature conditions [13,14]. However, to our knowledge, very few studies compare torsional resistance at different temperatures mimicking RT and BT. Therefore, this study aims to compare the torsional resistance of each heat-treated NiTi instrument for each temperature condition.

The null hypothesis is that there is no difference in mechanical properties (distortional angle, maximum torque and toughness) due to the phase transition that can be caused by temperature changes in each NiTi group.

## 2. Materials and Methods

### 2.1. Tested Nickel-Titanium Instruments

One conventional and three thermomechanically treated single-use NiTi rotary instrument systems, including OneShape (OS) (MicroMega, Besançon, France), OneCurve (OC) (MicroMega), WaveOne Gold (WOG) (Dentsply Maillefer, Ballaigues, Switzerland) and HyFlex EDM (HFE) (Coltene/Whaledent, Altstätten, Switzerland) were selected for this study (Table 1).

The instruments were divided into four experimental groups. Each group used 20 files from each instrument system, which were subdivided into 2 subgroups—room temperature (RT) and body temperature (BT) (*n* = 10/group)—based on different temperature conditions. The characteristics of the instruments were as follows:

Group 1: OS, which has a noncutting tip of size 25 and constant 6% taper. This instrument is made of a conventional austenite 55-NiTi alloy.

Group 2: OC, which has a noncutting tip of ISO size 25 and constant 6% taper. This instrument is composed of a NiTi alloy that undergoes a patent-protected heat treatment (C-wire), which provides a shape-memory effect and the capability of being precurved.

Group 3: WOG, which has a noncutting tip of ISO size 25 and 8% taper of a few millimeters. This instrument is manufactured using a new thermal process, the gold process, which is a postmanufacturing procedure in which the ground NiTi instrument is heat-treated and slowly cooled, making a superelastic NiTi instrument.

Group 4: HFE, which has a tip of size 25 and 8% taper of a few millimeters. This instrument is manufactured from controlled memory (CM) wire using EDM technology; a well-known noncontact machining procedure via electrical discharge machining.

### 2.2. Torsional Resistance Test

**Figure 1:**
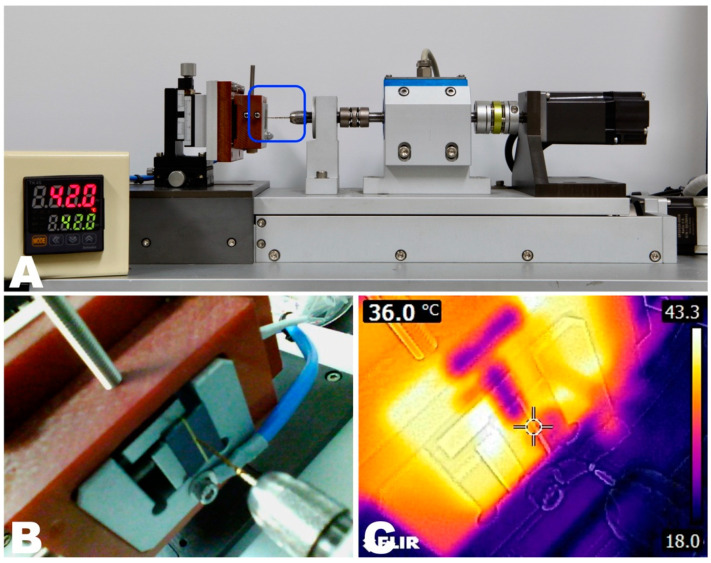
(**A**) Torsional resistance test device (AEndoS) used in this study. Electronic heat controller used to set the test temperatures. (**B**) The *Blue box* from A shows the instrument’s tip held between brass plates. (**C**) Real-time monitoring of the experimental temperature around the file tip.

The subdivided groups in each instrument system have two temperature conditions: RT (approximately 22 °C) and BT (maintained at 36 °C), which is estimated to be the temperature in the root canal. The temperature inside the root canal was set at 36 °C, as the temperature in the root canal has been reported to be 35.1 °C ± 1.0 °C [15]. To control the temperature, an electronic heat controller (TK4N/S/SP Autonics, Busan, Korea) was used to transfer heat three-dimensionally and directly to the brass plate where the instrument’s tip was held (Figure 1A,B). The temperature setting was confirmed immediately before each experiment using a thermal imaging camera (FLIR, FLIR Systems OU, Tallinn, Estonia) (Figure 1C).

AEndoS (DMJ system, Busan, Korea) was used to measure the parameters of torsional resistance (Figure 1), as described by Ha et al. [16]. Briefly, 3 mm of the instrument’s tip was secured between two brass plates while keeping the file straight. Then, it was rotated at a constant rotational speed (2 rpm) clockwise or counterclockwise, depending on its active direction, until it was fractured. During the instrument’s rotation, the torsional load (Ncm) and distortion angle (degrees) were recorded at a rate of 50 Hz. The toughness until fracture was computed from the area under the plot presenting distortion angle (*X*-axis) and torsional load (*Y*-axis) using Origin v6.0 Professional (Microcal Software Inc., Northampton, MA, USA).

### 2.3. Statistical Analysis

To evaluate the assumption of normality, the torsional resistance parameter was first analyzed using the Kolmogorov–Smirnov test. The main effect model of the two-way analysis of variance (ANOVA) test was used to analyze the relationship between temperature change and the instrument type for maximal torque, fracture angle and toughness at a significance level of 95% using SPSS Statistics 25 (IBM Corp., Somers, NY, USA). Additionally, to check the change in mechanical performance at two different temperatures for the same instrument, an individual *t*-test was performed.

### 2.4. Scanning Electronic Microscopic Analysis

After the test, to evaluate the topographic features of the fractured surfaces, all fractured fragments were observed using scanning electron microscopy (SEM) (SU8220, Hitachi High-Technologies Corporation, Tokyo, Japan).

### 2.5. Differential Scanning Calorimetry

Differential scanning calorimetry (DSC) analysis was performed to determine phase transformation temperature values. Five new specimens for each instrument system were chosen for DSC analysis (DSC Q2000 V24.4 Build 116, TA Instruments, New Castle, DE, USA), followed by the following process. First, it was cooled to −60 °C, and then, the temperature was maintained for 1 min. The sample was heated at a speed of 2 °C/min until it reached 100 °C. After a brief pause, the sample was cooled back to −60 °C in 2 °C/min. The heating and cooling curves were automatically acquired by DSC Q2000 (TA Instruments) to observe changes in the phase transition start temperature and enthalpy.

After performing the DSC analysis of the five NiTi instruments for each type, the phase transition temperature of each analysis was measured. The phase transition temperature analysis through DSC was performed according to ASTM F2004-1713. In the case of austenite starting temperature (A_s_), austenite finishing temperature (A_f_), R-phase starting temperature (R_s_), R-phase finishing temperature (R_f_), martensite starting temperature (M_s_) and martensite finishing temperature (M_f_), the graphical intersection of the baseline with the extension of the line of maximum inclination of the appropriate peak of the curve was measured and the peak temperatures for the concrete transformations of the endothermic and exothermic curves were measured [17,18,19,20].

## 3. Results

### 3.1. Torsional Resistance Test

The torsional resistance test results for each instrument are shown in Table 2. The results of the two-way ANOVA showed no significant difference between different temperature conditions (*p* < 0.05). The instrument system significantly influenced the parameters of torsional resistance. The HFE group showed the highest toughness, maximum torque and fracture angle values (*p* < 0.05).

When a *t*-test was conducted, only the distortional angle of OC showed a significant difference based on temperature, whereas other groups showed no significant differences in maximum torque, distortional angle and toughness between groups based on temperature (*p* < 0.05).

### 3.2. Differential Scanning Calorimetry

**Figure 2:**
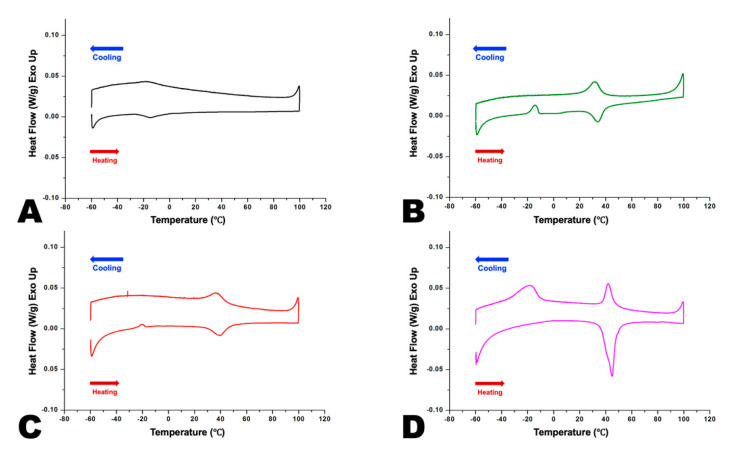
Differential scanning calorimetry (DSC) diagram from: (**A**) OneShape, (**B**) OneCurve, (**C**) WaveOne Gold and (**D**) Hyflex EDM NiTi instruments.

The DSC curves obtained from each NiTi instrument are shown in Figure 2. In the DSC diagram, the exothermic reaction in the upper curve indicates a martensitic transformation in the cooling process, whereas the endothermic reaction in the lower curve is caused by a reverse transformation from the martensitic to the austenitic phase in the heating process. On the cooling curve, there are two peaks: the first peak corresponds to the initial transformation from the austenite phase to the R-phase, and the second peak corresponds to the transformation from the R-phase to the martensite phase.

The phase transformation temperatures for each NiTi instrument are shown in Table 3. These transition temperatures can tell what phase each NiTi instrument exists in under each of the two experimental temperature conditions. Under both RT and BT conditions, the OS group were in a predominantly austenitic phase while the HFE group were in a martensitic phase. The OC and WOG groups were at predominantly martensitic phases at RT and a mix of martensitic and austenitic phases at BT. Based on the transformation peak temperature, more than half of the WOG was martensite, while the OC group was austenite in the BT condition.

### 3.3. Scanning Electronic Microscopic Analysis

**Figure 3:**
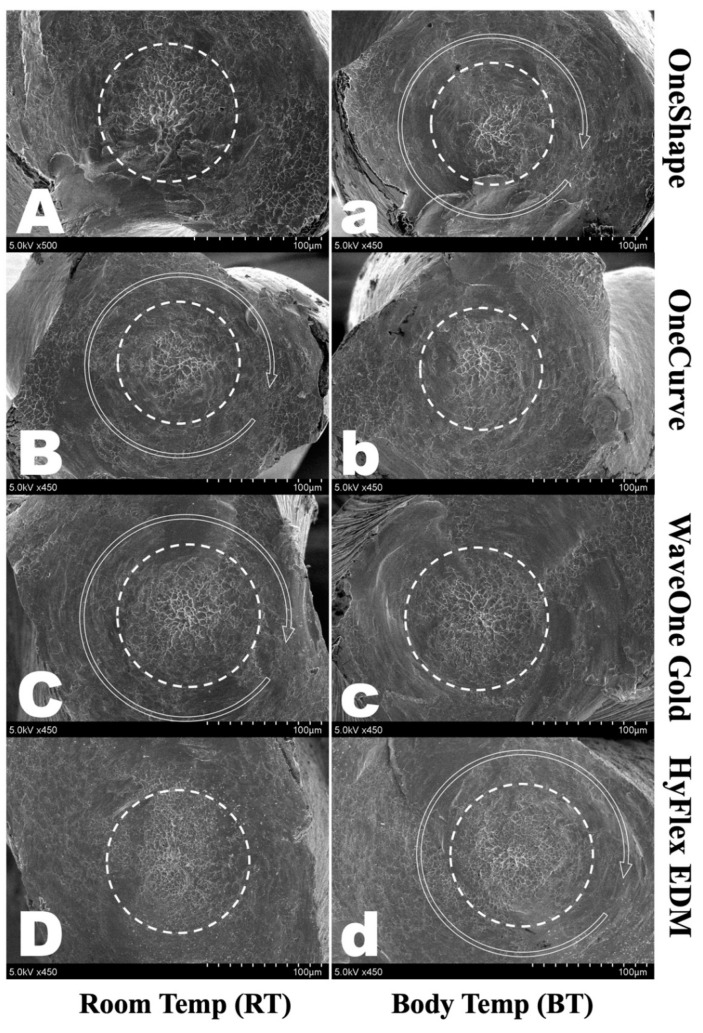
Scanning electron micrographs of the fracture surface of fractured segments after the torsional test. All showed typical features of torsional fractures, including concentric abrasion marks (*round arrow*) and fibrous dimples (*dotted circle*). OneShape (OS) (**A**) RT and (**a**) BT, OneCurve (OC) (**B**) RT and (**b**) BT, WaveOne Gold (WOG) (**C**) RT and (**c**) BT, HyFlex EDM (HFE) (**D**) RT and (**d**) BT.

SEM examination of the fractured cross-sectional surfaces revealed typical features of torsional fractures, concentric abrasion marks and fibrous dimples from the torsional center (Figure 3). Furthermore, no topographical difference was observed between instruments in different temperature groups.

## 4. Discussion

The torsional resistance test for NiTi instruments has mainly been performed according to ADA/ANSI specification No. 28 [21]. Furthermore, previous studies have attempted to apply test conditions to clinical situations [22,23]. Ha et al. evaluated the parameters of torsional resistance depending on the rotational speed and proved that the change of rotational speed did not result in a significant difference in torsional resistance under a clinically relevant rotational speed [22]. A repetitive torsional test was performed using a torque-control endodontic motor with a set of torque limits [24,25,26]. One study mimicked “brush-out motion” by enforcing lateral force on the instruments [2]. Kim et al. evaluated the effect of cyclic fatigue preloading on torsional resistance and pseudoelastic limit by repetitive torsional loading [23]. However, these studies did not specifically consider the BT condition in their tests.

Contemporary NiTi instruments are made using various heat-treatment technologies. Heat treatment plays an important role in the mechanical properties and clinical performance of NiTi instruments. The mechanical properties can be determined by the phase transformation temperature, which is inherited from the heat treatment method. The mechanical performance of NiTi instruments could be influenced by the differences between RT and BT. Various studies have reported the difference in cyclic fatigue resistance depending on temperature conditions. Testing cyclic fatigue at RT was regarded as having little clinical meaning and being outdated [27]. To the best of our knowledge, there are a lack of studies comparing torsional resistance at two different temperatures set at RT and BT. Therefore, this study aimed to compare the torsional resistance of each heat-treated NiTi instrument depending on temperature conditions.

Cunningham et al. [28] first assessed temperatures in human root canals in vivo in 1980. A digital microprobe thermometer was used to record the temperature inside teeth for different types of tooth. The temperature was measured in the range of 31 to 33.5 °C. The study also showed irrigant injected into the canal at room temperature reached an equilibrium with body temperature in 1 to 2 min. However, the finding was limited to the coronal part. De Hemptinne et al. [15] assessed temperature in the apical part of the root canal in vivo. Temperatures of irrigant were buffered rapidly to reach equilibrium at 35.1 °C (±1.0 °C) in the apical part. De Vaconcelos et al. [29] estimated that the intracanal temperature of the apical part where the NiTi files are used was around 35 °C in clinical conditions. Based on the results of de Hemptinne et al. [15] and de Vaconcelos et al. [29], the BT setting was determined for the present study.

In this study, four systems of NiTi instruments, made using different heat-treated methods, were selected. Depending on the results of the DSC analysis, we could speculate about the phase states of the instruments at the temperature that the instrument operates in the oral cavity. Since the manufacturer had not provided available data related to this phase transition, the phase states could be identified only through DSC analysis in this study [30,31]. The DSC analysis’ results indicated the phase state where the instrument is located, allowing the use of the right instrument in clinical practice [32].

Looking into the results of the torsional resistance test, no instruments showed a difference in torsional resistance depending on temperature conditions, except the distortional angle of OC. This implies that the change of phase composition did not influence the torsional resistance of the tested instruments, despite the fact that upregulation of the temperature from RT to BT conditions resulted in changing the instrument phase ratio. These results corresponded to those of previous studies that showed no significant decrease in torsional resistance regardless of heat-treatment technique but enhanced cyclic fatigue resistance by heat treatment [16,33,34]. Although the conventional OneFlare and heat-treated OneFlare exhibited different phase composition to each other at RT, they showed the same torsional resistance, but heat-treated OneFlare had higher cyclic fatigue resistance than that of conventional OneFlare [33]. R-phase heat-treated K3XF also showed no difference in torsional resistance but enhanced cyclic fatigue resistance by heat-treatment procedure [16]. A study by Silva et al. showed a similar result of temperature not affecting the torsional resistance of thermal treatment NiTi rotary instruments [34].

Among the four NiTi instruments, OC and WOG showed similar patterns of phase transition, except for the temperature that corresponds to the highest point of the peak temperature during austenite transformation (A_p_); because the A_s_ is lower than that of BT, and the A_f_ is higher than that of BT, their phases’ composition could be assumed to be a mix of austenite and martensite at BT. While the A_p_ of OC was lower than that of BT, the A_p_ of WOG was higher than that of BT. Since the A_s_ of OC is 25.3 °C on average and higher than that of RT (22 °C), the phase transition to austenite had begun, but it did not exceed the A_p_ (33.8 °C), so it could be estimated that martensite still existed as the dominant phase. However, at BT (36 °C), austenite would be the dominant phase because BT was beyond A_p_ (33.8 °C) and, subsequently, more than half of martensite had changed to austenite. Therefore, OC would act clinically in the same way as austenite. The A_s_ of WOG was 27.1 °C, and the predominant phase of that was martensite at RT (22 °C). Because the A_p_ of WOG was 38.4 °C, the martensite still existed as the dominant phase at BT (36 °C). Despite the different phase ratio in both WOG and OC at BT, there was no significant difference between their torsional resistances.

Because A_f_ of the OS was approximately −5 °C, the OS could exist in the austenite phase at both BT and RT. However, the A_s_ of HFE was above 38 °C, and it would exist as a totally flexible martensite when working in intraoral temperatures. Because OS and HFE have a single phase at both BT and RT, they did not show different torsional resistances.

When comparing torsional resistance between the instruments, HFE showed significantly higher results than other instruments. HFE is manufactured from CM wire and is the first endodontic instrument manufactured by the EDM process. EDM is a noncontact thermal erosion process used to machine electrically conductive materials through precisely controlled electrical discharges. The EDM process does not require direct contact with the NiTi wire, which eliminates mechanical stress from the traditional grinding process. Additionally, electrical sparks can cause local melting and partial evaporation of small portions of material. These small parts represent isotropic surface finishes which resemble craters. This surface treatment can be related to high torsional strength [35,36].

Under the limitations of this study, the torsional resistance of NiTi rotary instruments did not show a significant difference between BT and RT conditions. The different temperatures may be a more appropriate method to reproduce clinical conditions and may not change or increase the torsional fracture risk. This implies that clinicians can consider the cyclic fatigue resistance of heat-treated NiTi instruments in the BT condition without concern for torsional resistance. However, in clinical situations, not only is torsional resistance applied to the NiTi instrument but also cyclic fatigue is applied at the same time. This point was a limitation of this study. Therefore, further study is needed to determine whether these two types of fatigue are related to the phase transition when applied simultaneously.

## 5. Conclusions

The torsional resistance of the tested NiTi rotary instruments did not show a significant difference between the BT and RT conditions. Furthermore, the heat-treated NiTi rotary instruments may not change the torsional fracture risk at body temperature in a clinical setting.

## Figures and Tables

**Table 1:** NiTi rotary instrument systems tested in this study.

System (Code)	Heat-Treatment	Tip Size/Taper	Manufacturer
OneShape (OS)	conventional austenite alloy	ISO 25/Constant 6%	MicroMega, Besançon, France
OneCurve (OC)	C-wire	ISO 25/Constant 6%	MicroMega, Besançon, France
WaveOne Gold (WOG)	Gold-wire	ISO 25/Variable (8% taper of a few millimeters)	Dentsply-Sirona, Ballaigues, Switzerland
HyFlex EDM (HFE)	Controlled memory wirevia electrical discharge machining	ISO 25/Variable (8% taper of a few millimeters)	Coltene/Whaledent, Altstätten, Switzerland

**Table 2:** The results of torsional resistance (mean ± standard deviation).

	Temperature (°C)	Distortional Angle (Degree)	Toughness (Degree·Ncm)	Maximal Torque (Ncm)
OneCurve	RT 22 (°C)	439.4 ± 50.7 ^aA^	228.0 ± 75.4 ^aA^	0.781 ± 0.131 ^aA^
	BT (36 °C)	394.0 ± 41.0 ^bA^	216.1 ± 45.3 ^aA^	0.819 ± 0.126 ^aA^
OneShape	RT (22 °C)	439.4 ± 42.2 ^aB^	233.9 ± 47.0 ^aB^	0.751 ± 0.089 ^aB^
	BT (36 °C)	440.0 ± 97.6 ^aB^	236.2 ± 75.5 ^aB^	0.777 ± 0.145 ^aB^
Hyflex EDM	RT (22 °C)	582.9 ± 46.9 ^aC^	597.3 ± 71.6 ^aC^	1.583 ± 0.192 ^aC^
	BT (36 °C)	561.1 ± 53.1 ^aC^	539.8 ± 120.5 ^aC^	1.462 ± 0.233 ^aC^
WaveOne Gold	RT (22 °C)	389.3 ± 61.7 ^aD^	320-2 ± 103.3 ^aD^	1.194 ± 0.225 ^aD^
	BT (36 °C)	388.9 ± 63.6 ^aD^	309.0 ± 102.4 ^aD^	1.228 ± 0.142 ^aD^

There was no significant interaction effect between independent variables by 2-way analysis of variance (*p* > 0.05). ^A–D^; Different capital superscript letters indicate a significant difference among the different file systems in each distortional angle, toughness and maximal torque (*p* < 0.05). ^a,b^; Different lowercase superscript letters indicate a significant difference among the different temperatures in each instrument system (*p* < 0.05).

**Table 3:** Transformation temperatures for NiTi rotary instrument (mean ± SD, *n* = 5 for all instruments).

	Heating			Cooling					
	A_s_ (°C)	Peak	A_f_ (°C)	R_s_ (°C)	Peak	R_f_ (°C)	M_s_ (°C)	Peak	M_f_ (°C)
OneShape	−18.8 ± 4.0	−14.6 ± 0.3	−5.4 ± 4.4				−2.5 ± 0.9	−16.8 ± 0.3	−32.6 ± 2.5
OneCurve	25.3 ± 0.6	33.8 ± 0.2	40.3 ± 0.4				40.3 ± 0.4	31.5 ± 0.2	25.3 ± 0.6
WaveOne Gold	27.1 ± 2.6	38.4 ± 1.3	46.8 ± 0.1				44.8 ± 0.5	35.8 ± 1.0	22.2 ± 2.5
Hyflex EDM	38.1 ± 0.4	45.3 ± 0.7	48.1 ± 0.2	46.8 ± 0.1	41.9 ± 0.2	37.3 ± 0.3	−10.8 ± 0.5	−18.7 ± 0.6	−37.9 ± 0.5

## Data Availability

Data is contained within the article.

## References

[1] Shen Y., Zhou H.M., Wang Z., Campbell L., Zheng Y.F., Haapasalo M. (2013). Phase transformation behavior and mechanical properties of thermomechanically treated K3XF nickel-titanium instruments. J. Endod..

[2] Cho O.I., Versluis A., Cheung G.S., Ha J.H., Hur B., Kim H.C. (2013). Cyclic fatigue resistance tests of Nickel-Titanium rotary files using simulated canal and weight loading conditions. Restor. Dent. Endod..

[3] Cheung G.S., Oh S.H., Ha J.H., Kim S.K., Park S.H., Kim H.C. (2013). Effect of torsional loading of nickel-titanium instruments on cyclic fatigue resistance. J. Endod..

[4] Plotino G., Grande N.M., Testarelli L., Gambarini G. (2012). Cyclic fatigue of Reciproc and WaveOne reciprocating instruments. Int. Endod. J..

[5] Gambarini G., Gergi R., Naaman A., Osta N., Al Sudani D. (2012). Cyclic fatigue analysis of twisted file rotary NiTi instruments used in reciprocating motion. Int. Endod. J..

[6] Di Nardo D., Zanza A., Seracchiani M., Donfrancesco O., Gambarini G., Testarelli L. (2021). Angle of Insertion and Torsional Resistance of Nickel-Titanium Rotary Instruments. Materials.

[7] Loska S., Basiaga M., Pochrząst M., Łukomska-Szymańska M., Walke W., Tyrlik-Held J. (2015). Comparative characteristics of endodontic drills. Acta Bioeng Biomech..

[8] Shen Y., Zhou H.M., Zheng Y.F., Peng B., Haapasalo M. (2013). Current challenges and concepts of the thermomechanical treatment of nickel-titanium instruments. J. Endod..

[9] OneCurve Broucher. https://micro-mega.com/wp-content/uploads/2020/11/60301900-A_Flyer-One-Curve-Portfolio-EN_web.pdf.

[10] Gambarini G., Plotino G., Grande N.M., Al-Sudani D., De Luca M., Testarelli L. (2011). Mechanical properties of nickel-titanium rotary instruments produced with a new manufacturing technique. Int. Endod. J..

[11] Pereira E.S.J., Peixoto I.F.C., Viana A.C.D., Oliveira I.I., Gonzalez B.M., Buono V.T.L., Bahia M.G.A. (2012). Physical and mechanical properties of a thermomechanically treated NiTi wire used in the manufacture of rotary endodontic instruments. Int. Endod. J..

[12] Martins J.N.R., Silva E.J.N.L., Marques D., Belladonna F., Simões-Carvalho M., Vieira V.T.L., Antunes H.S., Braz Fernandes F.M.B., Versiani M.A. (2021). Design, metallurgical features, mechanical performance and canal preparation of six reciprocating instruments. Int. Endod. J..

[13] Dosanjh A., Paurazas S., Askar M. (2017). The Effect of Temperature on cyclic fatigue of nickel-titanium rotary endodontic instruments. J. Endod..

[14] Plotino G., Grande N.M., Testarelli L., Gambarini G., Castagnola R., Rossetti A. (2018). Cyclic fatigue of Reciproc and Reciproc Blue nickel-titanium reciprocating files at different environmental temperatures. J. Endod..

[15] de Hemptinne F., Slaus G., Vandendael M., Jacquet W., De Moor R.J., Bottenberg P. (2015). In vivo intracanal temperature evolution during endodontic treatment after the injection of room temperature or preheated sodium hypochlorite. J. Endod..

[16] Ha J.H., Kim S.K., Cohenca N., Kim H.C. (2013). Effect of R-phase heat treatment on torsional resistance and cyclic fatigue fracture. J. Endod..

[17] (2016). ASTM F 2004-17. Standard test method for transformation temperature of nickel-titanium alloys by thermal analysis. Am. Soc. Test. Mater.

[18] (2005). ASTM F 2005-21. Standard terminology for nickel-titanium shape memory alloys. Am. Soc. Test. Mater.

[19] (2018). ASTM E967-18. Standard test method for temperature calibration of differential scanning calorimeters and differential thermal analyzers. Am. Soc. Test. Mater..

[20] Kus K., Breczko T. (2010). DSC-investigations of the effect of annealing temperature on the phase transformation behavior in Ni-Ti shape memory alloy. Mater. Phys. Mech..

[21] (2008). ANSI/ADA Specification, No. 28-2008. Root Canal Files and Reamers, Type K.

[22] Ha J.H., Kwak S.W., Kim S.K., Sigurdsson A., Kim H.C. (2017). Effect from rotational speed on torsional resistance of the nickel-titanium instruments. J. Endod..

[23] Kim J.Y., Cheung G.S., Park S.H., Ko D.C., Kim J.W., Kim H.C. (2012). Effect from cyclic fatigue of nickel-titanium rotary files on torsional resistance. J. Endod..

[24] Park S.Y., Cheung G.S., Yum J., Hur B., Park J.K., Kim H.C. (2010). Dynamic torsional resistance of nickel-titanium rotary instruments. J. Endod..

[25] Ha J.H., De-Deus G., Versluis A., Kwak S.W., Kim H.C. (2019). Safe pseudoelastic limit range under torsional loading with Reciproc Blue. Int. Endod. J..

[26] Ha J.H., Sigurdsson A., De-Deus G., Versluis A., Kwak S.W., Kim H.C. (2018). Torsional Behavior of WaveOne Gold Endodontic File with the Dedicated Motor of the Original WaveOne File. Materials.

[27] Hulsmann M., Donnermeyer D., Schafer E. (2019). A critical appraisal of studies on cyclic fatigue resistance of engine-driven endodontic instruments. Int. Endod. J..

[28] Cunningham W.T., Balekjian A.Y. (1980). Effect of temperature on collagen-dissolving ability of sodium hypochlorite endodontic irrigant. Oral. Surg. Oral. Med. Oral. Pathol..

[29] de Vasconcelos R.A., Murphy S., Carvalho C.A., Govindjee R.G., Govindjee S., Peters O.A. (2016). Evidence for reduced fatigue resistance of contemporary rotary instruments exposed to body temperature. J. Endod..

[30] Brantley W.A., Svec T.A., Iijima M., Powers J.M., Grentzer T.H. (2002). Differential scanning calorimetric studies of nickel-titanium rotary endodontic instruments after simulated clinical use. J. Endod..

[31] Alapati S.B., Brantley W.A., Svec T.A., Powers J.M., Nusstein J.M., Daehn G.S. (2005). SEM observations of nickel-titanium rotary endodontic instruments that fractured during clinical Use. J. Endod..

[32] Alexandrou G.B., Chrissafis K., Vasiliadis L.P., Pavlidou E., Polychroniadis E.K. (2006). SEM observations and differential scanning calorimetric studies of new and sterilized nickel-titanium rotary endodontic instruments. J. Endod..

[33] Ataya M., Ha J.H., Kwak S.W., Abu-Tahun I.H., El Abed R., Kim H.C. (2018). Mechanical properties of orifice preflaring nickel-titanium rotary instrument heat treated using T-Wire technology. J. Endod..

[34] Silva E.J.N.L., Giraldes J.F.N., de Lima C.O., Vieira V.T.L., Elias C.N., Antunes H.S. (2019). Influence of heat treatment on torsional resistance and surface roughness of nickel-titanium instruments. Int. Endod. J..

[35] Pirani C., Iacono F., Generali L., Sassatelli P., Nucci C., Lusvarghi L. (2016). HyFlex EDM: Superficial features, metallurgical analysis and fatigue resistance of innovative electro discharge machined NiTi rotary instruments. Int. Endod. J..

[36] Uslu G., Ozyurek T., Yilmaz K. (2018). Comparison of alterations in the surface topographies of HyFlex CM and HyFlex EDM nickel-titanium files after root canal preparation: A three-dimensional optical profilometry study. J. Endod..

